# Are we really OHSS free?

**DOI:** 10.5935/1518-0557.20240040

**Published:** 2024

**Authors:** Garima Patel, Aryan Kashyap

**Affiliations:** 1 Consultant and Head, Reproductive Medicine, Ivy Hospital, Mohali, India; 2 Fellow, CIMAR The Women’s Hospital, Kochin, India

We read the recent article by Aziz *et al*. (2023) with great interest. Despite being a simple retrospective observational study, the study reinforces the idea that hyper-responder IVF patients must be stimulated with exogenous gonadotropins cautiously, using an antagonist protocol and a GnRH agonist trigger, to minimize the risk of OHSS (Devroey *et al*., 2011).

However, we, as readers, have specific queries regarding the study protocol and, in general, the hospital policies of the author, an answer to which will surely help us better understand the study.

1. E2 levels of more than 6000 pg/ml warrant using preventive measures for OHSS (Orvieto, 2013). However, in Table 1 of the article, we can see that the mean E2 level on the day of trigger in the hCG group was 13,081. We would like to ask the authors what measures were taken to prevent OHSS in these patients and why a GnRH agonist trigger was not used in the subset with high E2 levels on the day of trigger.

2. The current study shows a statistically significant difference between AFC and E2 levels on the day of trigger between the hCG and the GnRH agonist group. This difference seems to be the major drawback of the study since it is now well known that both AFC and E2 levels on the day of trigger directly impact the number of M-II oocytes and embryos obtained. The difference in AFC and E2 levels on the day of trigger between the two groups is a confounding factor and a source of bias in the current study.

3. According to the latest recommendation (ESHRE Clinic PI Working Group, 2021), the rate of cycles with moderate/severe OHSS in an antagonist cycle in high responders should not exceed 3 percent. However, the rate of OHSS in the hCG group in the current study has been quoted to be 11.8%, which is too high compared to the set competence levels. We ask the authors to audit the occurrence of OHSS at their center to rule out bias from the current study and also for the safety of their patients.

4. The current study does not mention whether the embryo transfer was done in fresh or frozen cycles. If we consider that a fresh transfer was done in the GnRH agonist group, it is not feasible to compare clinical pregnancy rates and ongoing pregnancy rates with the hCG groups since the GnRH agonist trigger has been associated with poor luteal phase support due to extensive luteolysis (Kolibianakis *et al*., 2005).

We appreciate the authors’ efforts to elucidate the difference in the embryological and reproductive parameters associated with hCG and the GnRH agonist trigger. Moreover, the current study is also a reminder for us to adopt the “OHSS free clinic” approach to preventing OHSS in IVF. An audit of OHSS rates done every three to six months can go a long way in helping us understand the fallacies of our treatment protocols and ensuring greater patient safety and satisfaction.
